# LncRNA SNHG16 promotes development of oesophageal squamous cell carcinoma by interacting with EIF4A3 and modulating RhoU mRNA stability

**DOI:** 10.1186/s11658-022-00386-w

**Published:** 2022-10-11

**Authors:** Lihua Ren, Xin Fang, Sachin Mulmi Shrestha, Qinghua Ji, Hui Ye, Yan Liang, Yang Liu, Yadong Feng, Jingwu Dong, Ruihua Shi

**Affiliations:** 1 Department of Gastroenterology, Zhongda Hospital, School of Medicine, Southeast University, 87 Dingjiaqiao Road, Nanjing, 210009 Jiangsu Province People’s Republic of China; 2 Department of Gastroenterology, Xuyi County People’s Hospital, Huaian, 211700 People’s Republic of China

**Keywords:** SNHG16, Oesophageal squamous cell carcinoma, mRNA stability, EIF4A3, RhoU

## Abstract

**Background:**

Numerous studies have revealed that long noncoding RNAs (lncRNAs) are closely related to the development of many diseases and carcinogenesis. However, their specific biological function and molecular mechanism in oesophageal squamous cell carcinoma (ESCC) remains unclear.

**Methods:**

RNA-Seq was performed to determine the differential expressions of lncRNAs in ESCC, and the level of SNHG16 expression was detected in ESCC and intraepithelial neoplasia (IEN) samples. In vitro and in vivo experiments were performed to explore the role of SNHG16 and the interaction of EIF4A3 and Ras homologue family member U (RhoU) signalling.

**Results:**

One hundred and seventy-five upregulated and 134 downregulated lncRNAs were identified by RNA-Seq. SNHG16 was highly expressed in ESCC and intraepithelial neoplasia (IEN) samples, and its expression level was correlated with tumour differentiation and T stage. Overexpression of SNHG16 can facilitate ESCC cell proliferation and metastasis. Mechanistically, we noticed that SNHG16 could bind RNA binding protein (RBP)-eukaryotic translation initiation factor (EIF4A3) and interact with it to form a complex. Importantly, the coalition of SNHG16 and EIF4A3 ultimately regulated Ras homologue family member U (RhoU). SNHG16 modulated RhoU expression by recruiting EIF4A3 to regulate the stability of RhoU mRNA. Knockdown of RhoU further alleviated the effect of the SNHG16 oncogene in ESCC cells.

**Conclusions:**

The newly identified SNHG16–EIF4A3–RhoU signalling pathway directly coordinates the response in ESCC pathogenesis and suggests that SNHG16 is a promising target for potential ESCC treatment.

**Supplementary Information:**

The online version contains supplementary material available at 10.1186/s11658-022-00386-w.

## Background

Oesophageal cancer (EC) is the third highest incidence cancer and the fourth highest cause of cancer-related mortality according to Cancer Statistics in China [1–3]. Approximately 90% of EC in China are oesophageal squamous cell carcinoma (ESCC) [2, 4], which shows aetiological and pathological characteristics distinct from oesophageal adenocarcinoma (EA) [5, 6]. Although the development of multimodality therapy has improved ESCC patient prognosis [7], the 5-year overall survival (OS) rate still remains unsatisfactory [8]. It is believed that late diagnosis and tumour metastasis propensity are associated with poor outcomes [3]. Genetic susceptibility, environmental factors and gene–environment interactions contribute to the development and progression of ESCC [9, 10]. An in-depth study of the molecular mechanisms of ESCC carcinogenesis and screening specific biomarkers are of particular significance for ESCC therapy and early diagnosis.

Long noncoding RNAs (lncRNAs), a recognized class of noncoding RNAs (ncRNAs) with lengths longer than 200 nucleotides (nt), have limited or no protein-coding capacity [11]. Although previous researchers regarded many lncRNAs as transcriptional noise, a growing number have been shown to have authentic biological functions such as chromatin modification, transcription, post-transcriptional regulation and nuclear transport [12–14]. As lncRNAs are more tissue specific or cell-type specific than protein-coding genes, they have distinct biological roles in physiological and pathological settings, especially in cancers [15]. Studies on and understanding of lncRNAs in ESCC carcinogenesis have gradually increased in recent years [16, 17]. Previous studies have discovered the expression profile of aberrant lncRNAs in ESCC, and identified varieties of ESCC-associated lncRNAs, some of which could be used as biomarkers for cancer diagnosis or prognosis [17–20]. Nevertheless, compared with the number of other cancer-associated lncRNAs, only very few ESCC-associated lncRNAs have been studied, and their functions and mechanisms have yet to be fully elucidated [20, 21]. Therefore, the vast majority of ESCC-associated lncRNAs need to be further investigated in detail.

In this study, we performed a next-generation RNA sequencing assay from four pairs of ESCC and normal oesophagus tissues to identify novel ESCC-associated lncRNAs. We then focused on a small nucleolar RNA host gene 16 (SNHG16) and detected its expression in a cohort of precancerous, cancerous and normal oesophageal tissues. In vitro and in vivo experiments were used to investigate the biological function of SNHG16. Finally, a mechanistic investigation was performed to determine how SNHG16 regulates ESCC cells, and explored its underlying targets.

## Materials and methods

### Patient sample collection and RNA-Seq screening

Oesophageal intraepithelial neoplasia (IEN) tissues, ESCC tissues and paired normal oesophagus tissues were obtained from inpatients who had previously received endoscopic submucosal dissection (ESD) or oesophagectomy with no chemoradiotherapy in the Department of Gastroenterology and the Department of Cardiothoracic Surgery at Zhongda Hospital Affiliated of Southeast University from February 2019 to November 2021. Staging of superficial neoplastic lesions of the oesophagus was done according to the Paris classification of gastrointestinal neoplasms. The protocol of this study complied with the ethical guidelines of Declaration of Helsinki principles and was authorized by the Ethics Committee of Zhongda Hospital (2019ZDSYLL022-P01).

Four pairs of ESCC tissues and normal oesophagus tissues were used to obtain a genome sequence screen (Kangchen Biotech, Shanghai, China). Detailed information is provided in Additional file 1: Table S1. Fold change (FC)/*P*-value/false discovery rate (FDR) filtration (multiple ≥ 1.5, *P* < 0.05 and FDR < 0.05) were determined to identify differentially expressed ESCC-related lncRNAs. Raw data are available on the Gene Expression Omnibus (GEO) website (GSE189830).

### Cell lines and culture

Four human ESCC cell lines (Eca109, KYSE30, KYSE140 and KYSE410) were purchased from the Institute of Biochemistry and Cell Biology of the Chinese Academy of Sciences (Shanghai, China). The normal human oesophageal epithelial cell line HET-1A was kindly provided by Professor Lin. L (The First Affiliated Hospital of Nanjing Medical University). Cells were cultured in RPMI 1640 medium (Gibco, Carlsbad, CA, USA) containing 10% fetal bovine serum (FBS, Gibco) at 37 °C in a humidified incubator supplemented with 5% CO_2_.

### Cell transfection and stable cell line establishment

Short hairpin RNA targeting SNHG16 (sh-SNHG16 #1/2/3) and small interfering RNA (siRNA) against EIF4A3 (si-EIF4A3 #1/2/3) and RhoU (si-RhoU #1/2/3) were synthesized by GenePharma (Shanghai, China) to knock down the respective gene expression. Cell transfection was conducted by Lipofectamine RNAiMAX (Invitrogen, Carlsbad, CA, USA) according to the manufacturer’s protocol. Stable cell lines were established by infection with the indicated lentiviruses and selected for puromycin (1–2 μg/ml, Sigma, MO, USA) resistance. Sequences of the the shRNA and siRNA used in this study are listed in Additional file 1: Table S2.

### RNA isolation, qRT-PCR and actinomycin D treatment

Total RNA from tissues and cells were extracted by an Omega Total RNA kit (Bio-Tek, USA). Reverse transcription was conducted based on the protocol of the Reverse Transcription kit (Takara, Tokyo, Japan). GAPDH and U6 were employed as normalization controls. The primers used in this study for qRT-PCR are listed in Additional file 1: Table S2.

To block transcription, 2 μg/ml actinomycin D (APExBIO, USA) was added to the cell culture medium after transfection. After actinomycin D co-culture for various time points, the remaining mRNA was detected by qRT-PCR.

### Western blot analysis and immunohistochemistry (IHC)

Western blotting was performed to analyse protein expression. The antibodies used were specific for EIF4A3 (1:1000; Abcam) and RhoU (1:500; Origene). GAPDH (1:6000, GeneTex) and β-actin (1:3000, GeneTex) were used as the controls. Protein bands were visualized using an enhanced chemiluminescence (ECL) chromogenic substrate (Beyotime, Shanghai, China) and assessed by Image-Lab analysis software (San Leandro, CA, USA).

Paraffin-block tissues of subcutaneous xenografts in mice were stained with haematoxylin and eosin (HE) and IHC, and subsequently evaluated by a pathologist blindly.

### Cell viability, colony formation, migration and wound healing assay

Cell viability was measured with a Cell Counting Kit-8 (CCK8) (Beyotime, Shanghai, China) at 24, 48, 72 and 96 h. Colony formation was performed by seeding 300–500 cells in a six-well plate. Two weeks later, the plates were washed and stained with crystal violet. Cell migration was determined by a Transwell assay and wound healing scratch assay according to standard protocols.

### Fluorescence in situ hybridization (FISH)

Cells were fixed with 4% paraformaldehyde and subsequently treated with 0.5% Triton X-100. For the FISH assay, a digoxigenin (DIG)-labelled SNHG16 probe (Service Bio, Wuhan, China) was used. Hybridization was conducted by utilizing a fluorescent using an in situ hybridization kit (RIBO Bio, Guangzhou, China) in a dark humidifying box at 37 °C overnight. The nuclei of cells were stained by 4′,6-diamidino-2-phenylindole (DAPI). Images were obtained by an Olympus confocal laser scanning microscope (Olympus Optical, Tokyo, Japan).

### Subcellular fractionation location

The separation of nuclear and cytosolic fractions was performed based on the protocol of the PARIS Kit (Life Technologies, Carlsbad, CA, USA). qRT-PCR was then used to determine the expression of SNHG16. U6 and GAPDH were used as internal controls for nuclear and cytoplasmic RNA.

### RNA protein pull-down assay and mass spectrometry (MS)

Sense and antisense of SNHG16 were transcribed in vitro with a T7 Quick High Yield RNA Synthesis Kit (Thermo Fisher Scientific, USA). Transcribed RNA was purified by RNA Clean & Concentrator-25 (Zymo Research, Beijing, China). A Pierce Magnetic RNA–Protein Pull-down kit (Thermo Fisher Scientific, MA, USA) was used according to the manufacturer’s instructions. After RNA pull-down, equal amounts of samples pulled down by sense and antisense SNHG16 were loaded on SDS–PAGE gels. Then, the gel was stained with a Protein Fast Silver Stain Kit (Leagene, Beijing, China) according to the protocol. Bands were cut and analysed by liquid chromatography–mass spectrometry (LC–MS/MS) (Oebiotech Company, Shanghai, China). Protein identifications were retrieved from the human RefSeq protein database (National Center for Biotechnology Information) using Mascot version 2.4.01 (Matrix Science, London, UK).

### RNA immunoprecipitation (RIP) assay

RIP was implemented with the Imprint RNA Immunoprecipitation (RIP) Kit (Sigma, Aldrich, US). Cell extracts were obtained by RIPA lysis buffer and then incubated with a mixture of magnetic beads and antibodies against EIF4A3. Anti-IgG antibody was utilized as a control. The final co-precipitated RNAs were purified and subjected to RT-PCR or qPCR.

### In vivo xenograft tumour models

Female BALB/c nude mice (4–6 weeks of age, GemPharmatech Co. Ltd, Nanjing, China) received humane care according to the Guide for the Care and Use of Laboratory Animals, and were raised under specific pathogen-free conditions. A total of 5 × 10^6^ SNHG16 stable knockdown or overexpressed cells and control cells were subcutaneously injected into the back of each mouse. Tumour size was calculated as (length × width^2^)/2 and recorded every 3 days. Mice were sacrificed 30–40 days after injection, and subcutaneous tumours were obtained and imaged. The animal study was approved by the institutional review board of Southeast University (20210709006) and was performed in compliance with the Basel Declaration.

### Statistical analysis

Experiments in our study were carried out in triplicate, and the values are expressed as the mean ± standard deviation (SD). Statistical significance was determined by the nonparametric Mann–Whitney *U* test or two-tailed paired Student’s *t*-test. The findings were considered to be significant at *P*  < 0.05. Statistical analyses were performed using SPSS 22.0 software (IBM, NY, USA) and GraphPad Prism v5.01 (GraphPad, La Jolla, CA, USA).

## Results

### SNHG16 expression is significantly upregulated in ESCC and IEN tissues

**Fig. 1:**
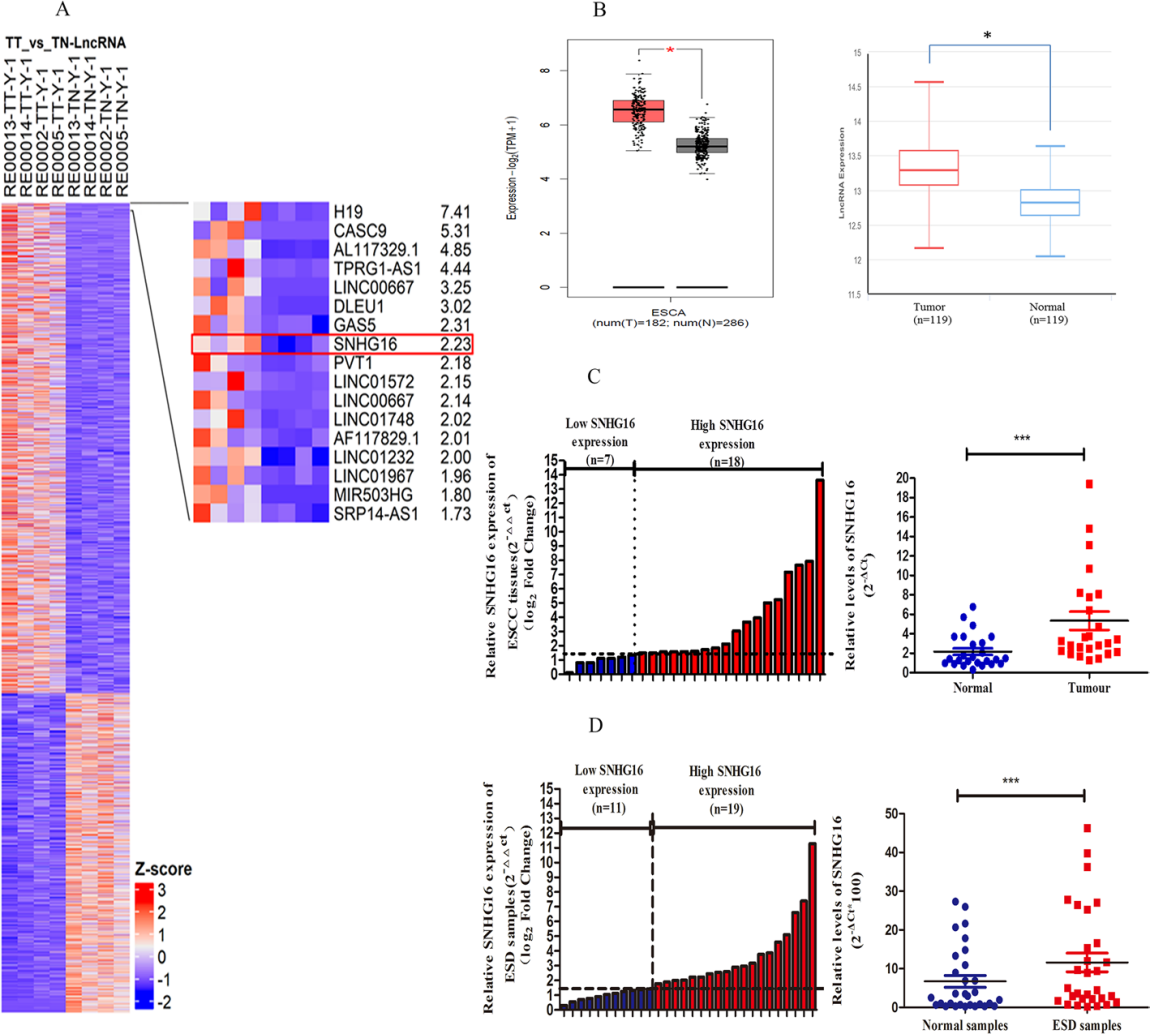
Relative SNHG16 expression in ESCC and IEN tissues and its clinical significance. **A** Hierarchical cluster heatmap of the differentially expressed lncRNAs in RNA-Seq analysis of four paired ESCC and adjacent tissues. Each row represents a gene, and each column represents a sample. Red represents upregulation of genes, and blue represents downregulation of genes. SNHG16 was one of the upregulated genes from next-generation RNA sequencing. TT, tumour tissue; TN, paired normal tissues. **B** SNHG16 expression in a larger independent cohort of 301 ESCC samples and 405 normal samples from the TCGA database (GSE53624, 53622). **C** SNHG16 expression was examined by qRT-PCR and normalized to GAPDH in 25 paired human ESCC tissues. **D** SNHG16 expression was examined by qRT-PCR and normalized to GAPDH in 30 pairs of ESD samples (*P < 0.05, **P < 0.01, ***P < 0.001)

RNA sequences detected 2145 different lncRNAs, including 175 upregulated (fold change > 1.5, *P* < 0.05) and 134 downregulated (fold change < 0.5, *P* < 0.05). Here, we focus on SNHG16, for which no function in ESCC has been previously ascribed. Among all the differentially expressed lncRNAs, SNHG16 was one of the upregulated lncRNAs in ESCC tissues (Fig. 1A). Two publicly accessible microarray datasets, including 301 oesophageal carcinoma samples and 405 normal samples, also verified the higher level of SNHG16 expression (GSE53624, 53,622) (Fig. 1B). Furthermore, we examined SNHG16 expression in 25 primary ESCC tissues and matched normal oesophageal tissues, and found that SNHG16 was significantly upregulated in ESCC tissues (Fig. 1C). We further detected SNHG16 in samples resected by ESD therapy. To our surprise, SNHG16 expression was high in 19 and low in 11 of 30 ESD samples (Fig. 1D), and its expression was correlated with tumour differentiation (*P* = 0.037) and T stage (*P* = 0.098), suggesting that SNHG16 upregulation was an early event in ESCC development. The correlation between SNHG16 expression and detailed clinical baseline characteristics of the patients in our study are presented in Table 1.

**Table 1:** Correlation between SNHG16 expression and clinical pathological characteristics of ESCC patients

Characteristics	All cases (%)	SNHG16 expression		*P* value
		High-expression cases (*n*)	Low-expression cases (*n*)	
Total number	55			
Gender				
Male	38	28	10	0.033
Female	17	7	10	
Age				
≥ 60	50	31	19	0.643
< 60	5	4	1	
Degree of tumour differentiation				
Cancer	42	30	12	0.037
HG-IEN	3	2	1	
LG-IEN	10	3	7	
Tumour location				
Upper/middle	30	20	10	0.779
Lower	25	15	10	
Tumour length				
≥ 3 cm	41	28	13	0.335
< 3 cm	14	7	7	
T stage				
T0	10	3	7	0.098
Tis	3	2	1	
T1	17	13	4	
T2	22	14	8	
T3	3	3	0	
Lymphatic metastasis				
Positive	7	6	1	0.402
Negative	48	29	19	

*HG-IEN* high-grade intraepithelial neoplasia, *LG-IEN* low-grade intraepithelial neoplasia

### SNHG16 promotes ESCC cell proliferation and migration in vitro

**Fig. 2:**
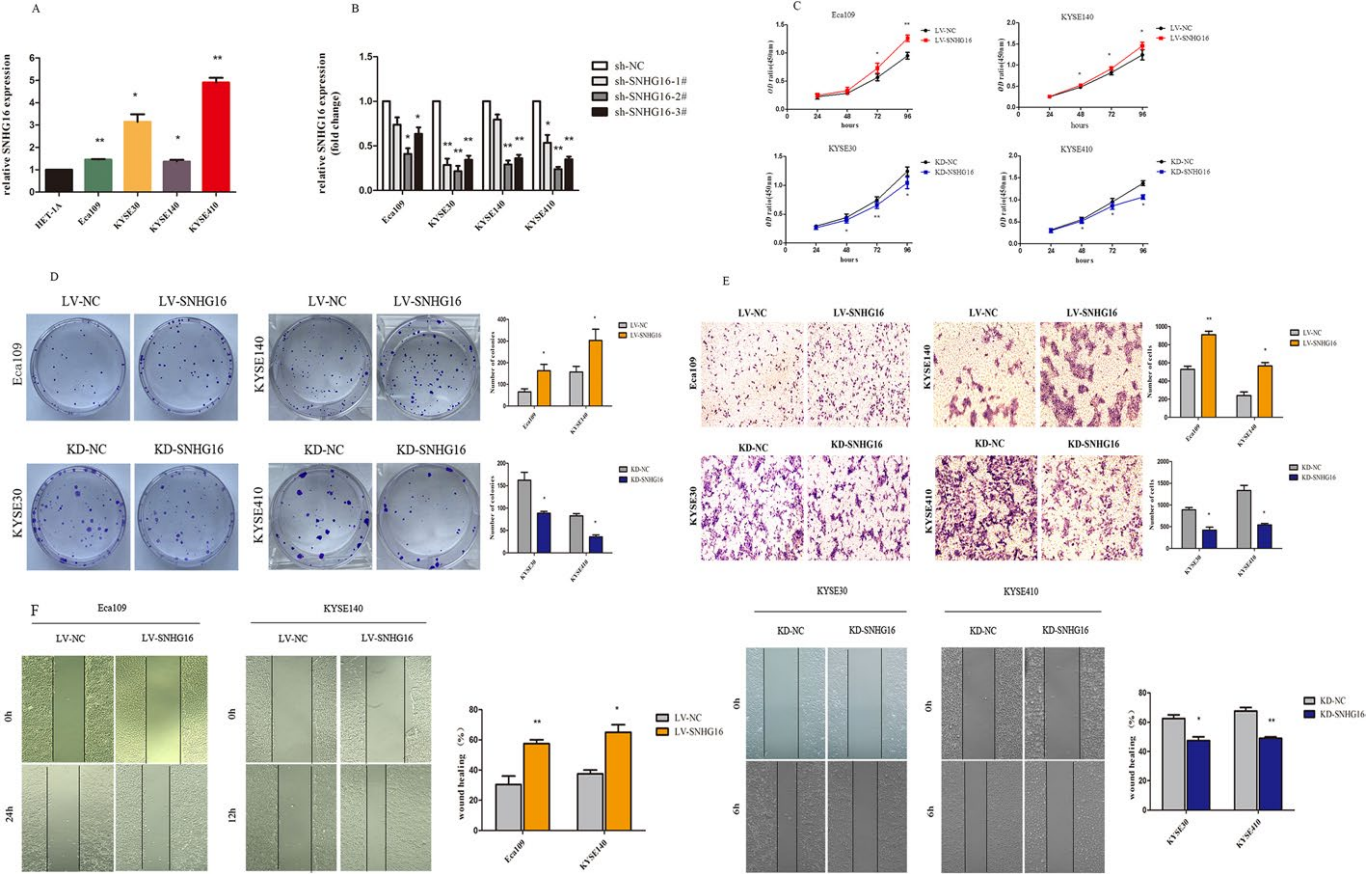
The effect of SNHG16 on ESCC cell proliferation and migration in vitro. **A** The relative expression of SNHG16 in the normal human oesophageal epithelium cell line (HET-1A) and four ESCC cell lines (Eca109, KYSE30, KYSE140 and KYSE410). **B** Relative expression levels of SNHG16 in four ESCC cell lines transfected with sh-NC or sh-SNHG16 #1, #2 and #3 tested by qRT-PCR. **C** The effects of SNHG16 on ESCC cell proliferation ability, colony formation ability (**D**) and migration ability from Transwell assays (**E**) and wound healing assays (**F**). Error bars indicate the mean ± s.e.m. from three independent experiments (*P < 0.05, **P < 0.01, ***P < 0.001)

To explore the biological role of SNHG16 in ESCC, we detected SNHG16 expression in diverse human ESCC cell lines. As shown in Fig. 2A, the level of SNHG16 was increased in ESCC cell lines (Eca109, KYSE30, KYSE140 and KYSE410) compared with the normal oesophageal cell line (HET-1A). Three independent shRNAs, #1, #2 and #3, were transfected into ESCC cell lines to knockdown SNHG16 expression. It was satisfactory that SNHG16 was more efficiently diminished by shRNA #2, which was selected for the primer sequence of the lentivirus package (Fig. 2B). As endogenous SNHG16 levels in KYSE30 and KYSE410 cells were higher than those in Eca109 and KYSE140 cells, they became stable cell lines packaged with lentivirus to stably knockdown SNHG16. Additionally, SNHG16 was ectopically overexpressed with a lenti-SNHG16 vector in Eca109 and KYSE140 cells.

Cell proliferative vitality was analysed by CCK-8 and colony formation assays, which showed that knockdown of SNHG16 decreased cell proliferation and colony formation compared with scrambled control cells (Fig. 2C, D). To estimate whether SNHG16 could affect ESCC cell migration, Transwell and scratch wound healing assays were performed. Interestingly, the number of migrating KYSE30 and KYSE410 cells was strongly reduced after SNHG16 knockdown (Fig. 2E). Scratch wound healing assays also showed that SNHG16 knockdown significantly suppressed migration ability (Fig. 2F). shRNA depletion experiments may suffer from off-target effects, therefore, we subsequently investigated the effect of SNHG16 overexpression. As shown in Fig. 2C–F, Eca109 and KYSE140 cells overexpressing SNHG16 displayed more cell proliferation and migration ability than controls. Altogether, gain- and loss-of-function experiments showed that SNHG16 promotes ESCC cell proliferation and migration in vitro.

### SNHG16 promotes ESCC cell tumourigenesis in vivo

**Fig. 3:**
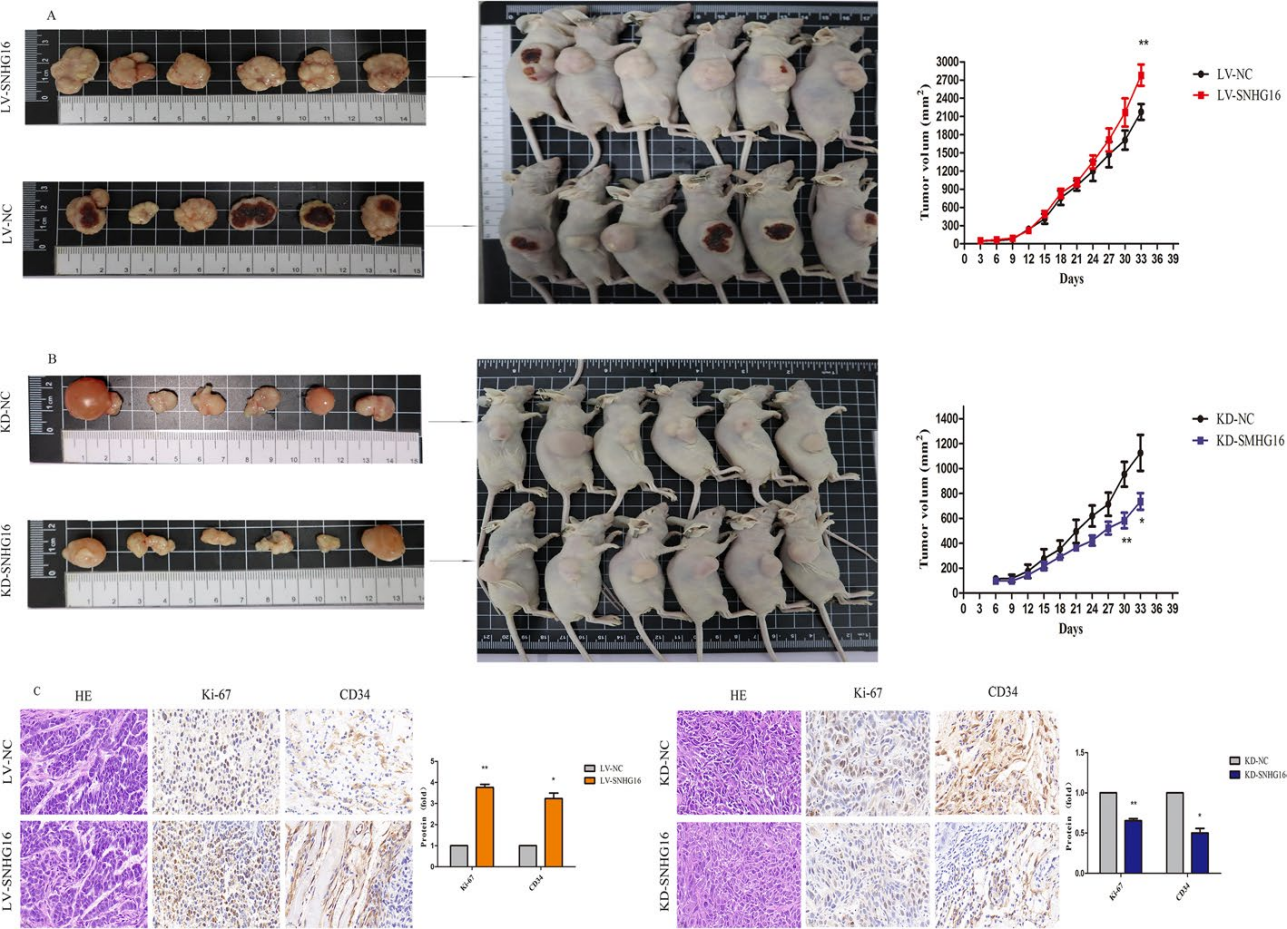
The effect of SNHG16 on ESCC cell tumourigenesis in vivo. Images of the xenograft model in BALB/c nude mice from four treatment groups (*n* = 6 for each group) on day 35. **A** Images of mice and tumour volumes of LV-SNHG16 and the control group (LV-NC). **B** Tumour volumes and growth curve of KD-SNHG16 and the control group (KD-NC). **C** Tumour sections under HE staining and IHC staining for Ki-67 and CD34. Scale bar, 100 μm. Data are listed as the mean ± SD of three independent experiments. *P < 0.05, **P < 0.01, ***P < 0.001

To further clarify if SNHG16 exerts ESCC cell carcinogenesis effects in vivo, Eca109 cells stably overexpressing SNHG16 or control empty vector were subcutaneously injected into nude mice. As displayed in Fig. 3A, tumour volumes and tumour growth in the LV-SNHG16 group was obviously faster than in the control group in the whole process of feeding. After 35 days, ultimate xenograft tumours were obtained. The xenograft tumours were photographed and showed that tumours formed in the control group were generally smaller than those in the LV-SNHG16 group (Fig. 3A). In contrast, the tumour weight and tumour growth after injection with KD-SNHG16 were smaller than those of the negative control groups (Fig. 3B). We further detected the staining of Ki-67 and CD34 through immunohistochemistry (IHC) analysis, which showed higher Ki-67 and CD34 intensity in the LV-SNHG16 group than in the control group, while crosscurrent results were observed in the KD-SNHG16 group (Fig. 3C).

### SNHG16 interacts with EIF4A3

**Fig. 4:**
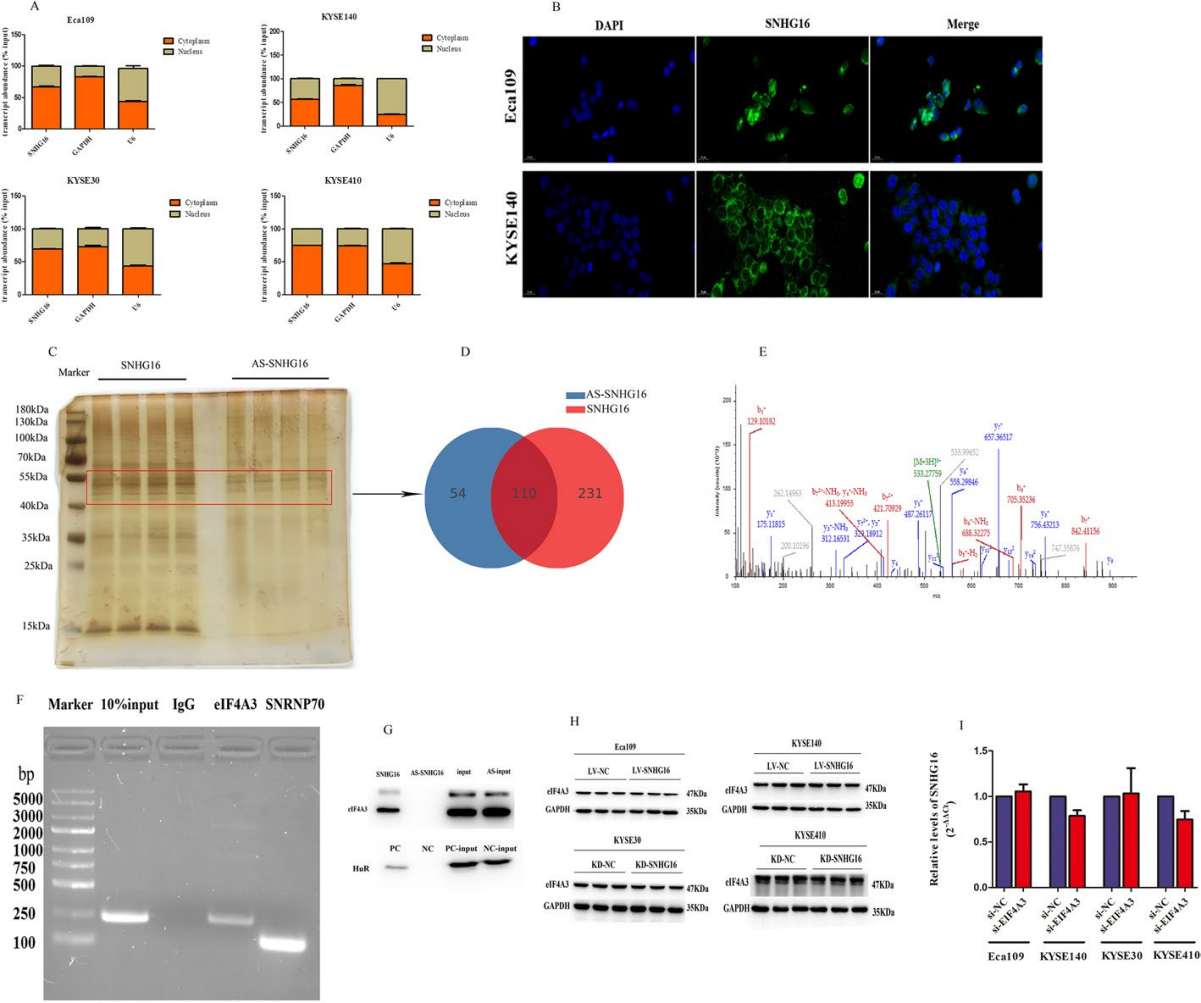
SNHG16 interacts with EIF4A3. SNHG16 expression in the cell nucleus or cytoplasm of ESCC cells, as detected by subcellular fractionation (**A**) and FISH (**B**) assays (scale bar, 100 μm). (**C**) Pull-down silver assay showed differential RBPs interacting with SNHG16 and antisense SNHG16. **D** LC–MS identified the differential RBPs binding to SNHG16 and anti-SNHG16. **E** The peptide sequence of EIF4A3, followed by LC–MS, specifically interacted with SNHG16. RIP **F** and Western blot **G** after pull down assays further validated the interaction between EIF4A3 and SNHG16. **H** Western blot analysis showed the expression of EIF4A3 after SNHG16 overexpression or downregulation. **I** qRT-PCR showed the expression of SNHG16 after EIF4A3 knockdown. *P < 0.05, **P < 0.01, ***P < 0.001

To further demonstrate the potential molecular mechanisms of SNHG16 in ESCC, subcellular fractionation and FISH staining were used. As displayed in Fig. 4A, B, SNHG16 was mostly located in the cytoplasm against the nucleus, which suggested that SNHG16 may play a regulatory role at the post-transcriptional level. Pull-down assays were carried out to identify the probable RNA binding proteins (RBPs) binding to SNHG16. Intriguingly, Fig. 4C showed specific bands in the 40–55 kDa, 35 kDa and 15 kDa regions by silver staining, which implies that some RBPs bind to SNHG16. Considering that the range of 40–55 kDa can pull down more proteins, we performed LC–MS from this band to look for specific RBPs interacting with SNHG16. Subsequently, we discovered 341 differential RBPs binding to SNHG16 and 164 RBPs binding to antisense SNHG16, according to LC–MS (Fig. 4D). We stripped away the overlapping proteins and focused on EIF4A3, which specifically binds to SNHG16 (Fig. 4E). As shown in Fig. 4F, the abundance of SNHG16 was detected in the precipitates of EIF4A3 antibody, and the enrichment of EIF4A3 protein in the products pulled down by SNHG16 (Fig. 4G) further confirmed the results of LC–MS. Dramatically, we noticed that the level of EIF4A3 remained unaltered after SNHG16 knockdown or overexpression (Fig. 4H), and SNHG16 exhibited no significant change with EIF4A3 depletion (Fig. 4I), which suggests that SNHG16 and EIF4A3 are not downstream regulators of each other but may form a complex. Altogether, SNHG16 may recruit EIF4A3 to regulate its downstream genes.

### EIF4A3 acts as an oncogene in ESCC cell progression

The online GEPIA database (http://gepia.cancer-pku.cn/) shows that EIF4A3 is highly upregulated in oesophageal cancer, and its expression is positively correlated with SNHG16 (Additional file 1: Fig. S1A). To verify its role in ESCC, we synthesized siRNAs target EIF4A3 to knock down its expression. As shown in Additional file 1: Fig. 1B, si-EIF4A3 #1 exhibited ~ 50% knockdown efficiencies and was selected for subsequent functional experiments. CCK-8 and colony formation assays showed that EIF4A3 downregulation significantly reduced ESCC cell viability (Additional file 1: Fig. S1C-D). In addition, cell migration ability was also weakened due to EIF4A3 knockdown according to the transwell and scratch wound healing assays (Additional file 1: Fig. S1E–F). These findings indicated that EIF4A3 promoted ESCC cell malignant proliferation and migration, corresponding with the oncogenic role of SNHG16 in ESCC.

### SNHG16–EIF4A3 regulates RhoU and affects its mRNA stability

**Fig. 5:**
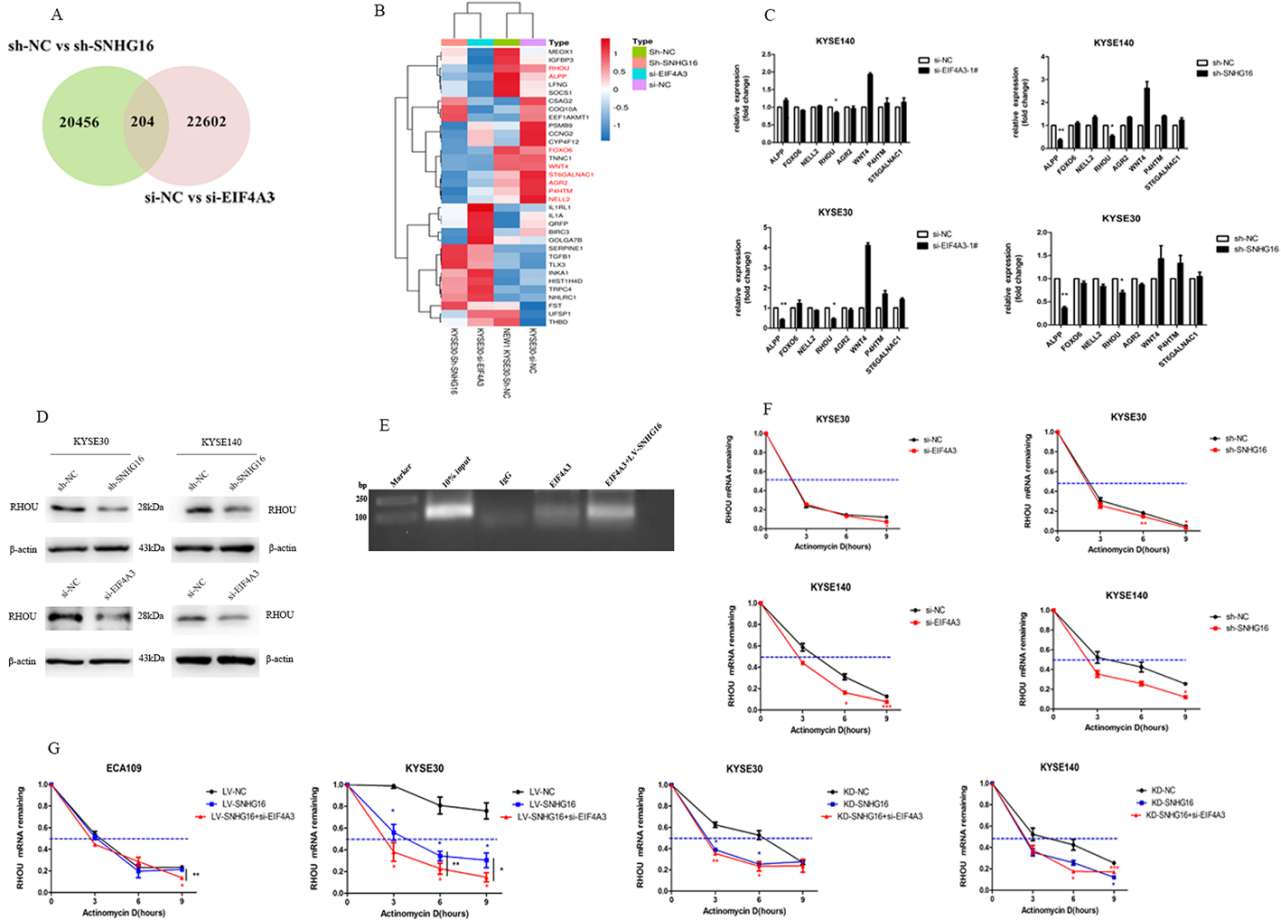
SNHG16–EIF4A3 regulates RhoU and affects its mRNA stability. **A** A total of 204 transcripts were altered simultaneously in sh-SNHG16-treated cells and si-EIF4A3-treated cells (fold-change > 1.5, *P* < 0.05). **B** The hierarchical cluster heatmap of the 204 differentially expressed mRNAs in RNA-Seq analysis of sh-SNHG16-treated cells and si-EIF4A3-treated cells. Red represents upregulated transcripts, and blue represents downregulated transcripts. **C** qRT-PCR validated the changes in eight mRNAs involved in the regulation of sh-SNHG16 and si-EIF4A3. **D** Western blot analysis of RhoU after SNHG16 or EIF4A3 downregulation in ESCC cells. GAPDH was used as an internal control. **E** RIP showed that EIF4A3 interacted with RhoU and increased under SNHG16. **F** RNA stability assays were performed to measure the degradation rates of RhoU mRNA in KYSE30 and KYSE140 cells with SNHG16 or EIF4A3 knockdown. **G** RhoU mRNA stability in ESCC cells with LV-SNHG16 cells, KD-SNHG16 cells, and EIF4A3 knockdown in LV-SNHG16-treated cells and KD-SSNHG16 cells. *P < 0.05, **P < 0.01, ***P < 0.001

To investigate the associated signal pathways and potential target genes involved in SNHG16–EIF4A3 regulation in ESCC, a high-resolution transcriptome microarray (Shengyin Biotech, Shanghai, China) after SNHG16 or EIF4A3 knockdown in ESCC cells was performed. Microarray analysis identified 20,456 differentially expressed genes after SNHG16 knockdown, and 22,602 altered genes after EIF4A3 knock down (fold change > 1.5, *P* < 0.05). Notably, we found 204 genes at the overlap of these two gene sets, which were indicated to be the co-targets regulated by SNHG16 and EIF4A3 (Fig. 5A). The mRNA levels of RhoU, FOXO6, WNT4, ST6GALNAC1, AGR2, P4HTM, NELL2 and ALPP were significantly downregulated due to SNHG16 or EIF4A3 knockdown in ESCC cells according to RNA-Seq (Fig. 5B). To further validate the screen common target mRNAs, we performed qRT-PCR to explore the regulation of the eight mRNA by SNHG16 and EIF4A3. As displayed in Fig. 5C, only RhoU mRNA was significantly diminished in both KYSE30 and KYSE140 cells when SNHG16 or EIF4A3 was knocked down. In addition, the protein level of RhoU was significantly reduced after SNHG16 or EIF4A3 silencing (Fig. 5D). Furthermore, the results of RIP assay showed that EIF4A3 could interact with RhoU, and the enrichment of RhoU in anti-EIF4A3 precipitates was strengthened due to SNHG16 overexpression (Fig. 5E).

Previous studies have reported that lncRNAs and RBPs can regulate mRNA stability, so we treated KYSE30 and KYSE140 cells with actinomycin D, which measures the decay of pre-existing mRNA. As shown in Fig. 5F, downregulation of SNHG16 or EIF4A3 decreased the RhoU mRNA half-life. Co-silencing SNHG16 and EIF4A3 in ESCC cells further decreased RhoU mRNA stability. However, the upregulation of SNHG16 did not strengthen RhoU mRNA stability (Fig. 5G**)**. To illustrate whether the function of SNHG16 was dependent on EIF4A3, we further transfected LV-SNHG16 cells with si-EIF4A3. As expected, EIF4A3 knockdown decreased RhoU mRNA stability in LV-SNHG16 cells, which implied a more dominant role in regulating mRNA stability (Fig. 5G). Altogether, these results implied that SNHG16 modulated RhoU expression by recruiting EIF4A3 to enhance RhoU mRNA stability.

### RhoU is involved in the oncogenic role of SNHG16

**Fig. 6:**
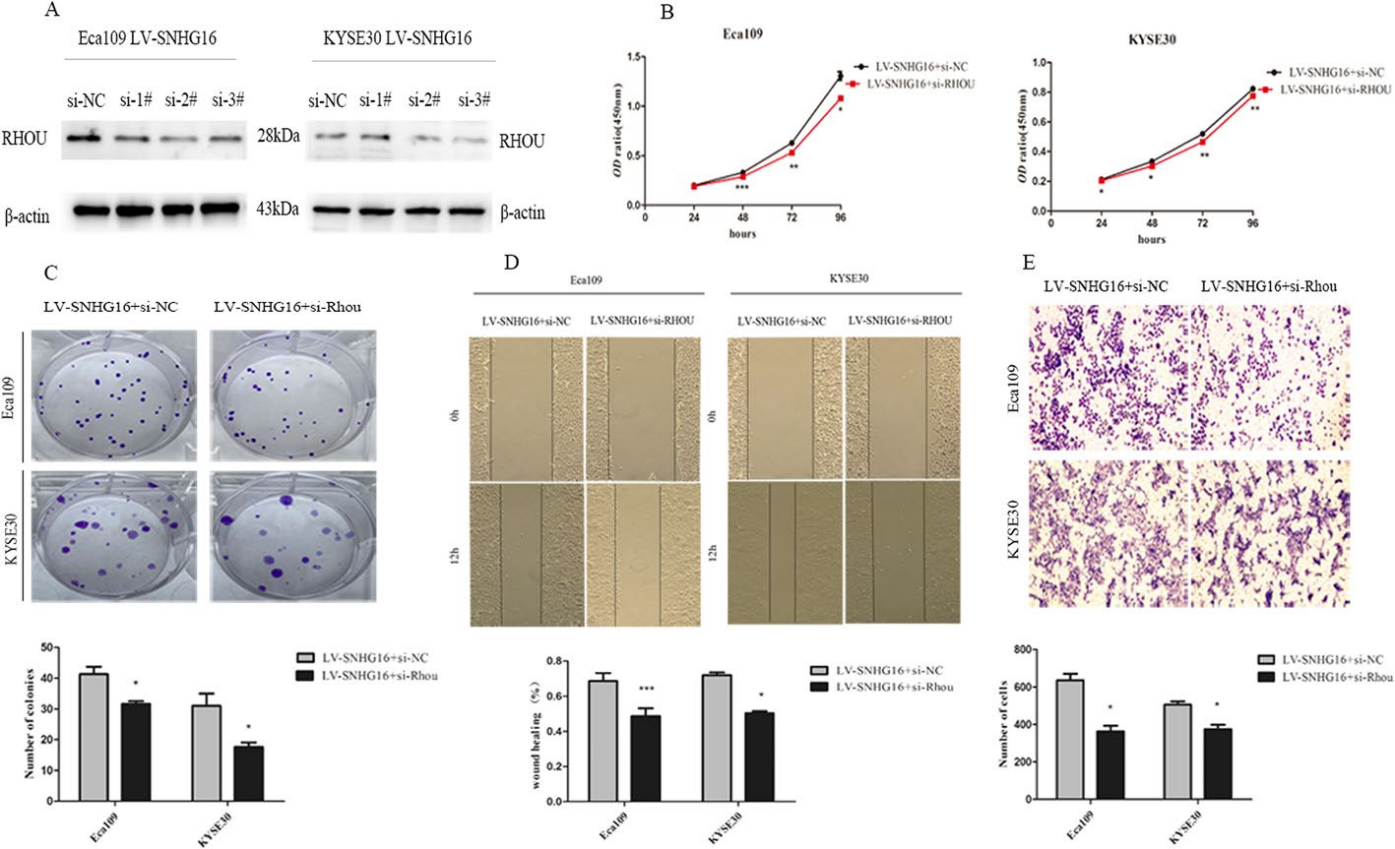
RhoU is potentially involved in the oncogenic function of SNHG16. **A** The interfering efficacies of si-RhoU-1#, 2# and 3# measured by Western blot. β-actin was used as an internal control. **B** CCK-8 and colony formation **C** assays were performed to evaluate the effects of RhoU combined with SNHG16 on Eca109 and KYSE30 cell proliferation. **D** Wound healing and Transwell assays **E** were performed to validate the effects of RhoU combined with SNHG16 on the migration ability of Eca109 and KYSE30 cells. *P < 0.05, **P < 0.01, ***P < 0.001

To explore whether RhoU was involved in SNHG16-induced ESCC cell proliferation and migration, we carried out rescue experiments. Figure 6A shows that siRNA #1, #2 and #3 could not adequately inhibit inhibition individually, so we used siRNA #1, #2 and #3 by using the smart pool method to transfect ESCC cells in the following functional assays to achieve more effective RhoU inhibition. Fortunately, CCK-8 and colony formation assays showed that cell proliferation was increased by SNHG16 overexpression and was repressed when RhoU was knocked down (Fig. 6B, C). Similarly, cell migration ability was impaired after RhoU suppression (Fig. 6D, E). Rescue experiments showed that SNHG16 partially affected the tumourigenesis and development of ESCC through RhoU.

## Discussion

Long noncoding RNAs (lncRNAs) have been recently identified as key participators in cancer-related biological processes, and indicate novel molecular targets in cancer. Herein, we performed a high-throughput analysis of ESCC tissues and normal oesophageal tissues and discovered 2145 aberrantly expressed lncRNAs, which updates the current ESCC lncRNA profile. We first reported high SNHG16 expression in high-grade intraepithelial neoplasia (HG-IEN) and showed that its expression was correlated with tumour differentiation and T stage, which indicates that it may play an oncogenic role in early ESCC. Mechanistically, we acknowledge that SNHG16 could bind to some RBPs. Furthermore, we targeted eukaryotic translation initiation factor (EIF4A3), a core component of exon junction complex, and demonstrated that the SNHG16–EIF4A3 coalition ultimately regulated the expression of the Ras homolog family member U (RhoU). SNHG16 modulated RhoU expression by recruiting EIF4A3 to enhance the mRNA stability of RhoU. In conclusion, our data suggest that the SNHG16–EIF4A3–RhoU axis might provide new insight into the mechanism underlying ESCC development.

Small nucleolar RNA host gene 16 (SNHG16), located on 17q25.1, was first reported in aggressive neuroblastoma [22]. Subsequent studies identified its oncogenic role in other cancers, such as lung [23], cervical [24], breast [25] and colorectal cancers [26]. However, its role in hepatocellular cancer is controversial [27, 28], which may be attributed to tissue-specific or cell type-specific properties. Recently, SNHG16 expression was shown to be upregulated in ESCC tissues compared with normal tissues [29, 30], indicating its oncogenic effect in ESCC. In this study, we noticed that SNHG16 was upregulated in ESCC tissues, according to our microarray analysis. We further explored its expression in a large cohort of ESCC tissues and confirmed its upregulation in ESCC tissues and ESCC cell lines.

Currently, ESCC tends to be detected at an early stage because of the prevalence of upper gastrointestinal endoscopy screening [31]. Endoscopic submucosal dissection (ESD) has become an alternative, minimally invasive strategy to oesophagectomy, especially for oesophageal intraepithelial neoplasia (IEN) [32]. The detection of SNHG16 in IEN can help us to better understand its role in the tumourigenesis of ESCC. Here, we collected 30 pairs of oesophageal neoplasia samples (obtained by ESD therapy) and first found that the level of SNHG16 was high in 19 T1N0M0 patients, which indicates that SNHG16 is an ESCC-related biomarker. Furthermore, in vitro and in vivo assays showed that ectopic expression of SNHG16 prompted ESCC cell proliferation and migration ability. These findings imply an oncogenic role of SNHG16 in the development and progression of ESCC.

Accumulating evidence suggests that lncRNAs promote or suppress cancer by sponging miRNAs. It was originally reported that SNHG16 has 27 AGO/miRNA binding sites along its full length, indicating that SNHG16 might function as a competing endogenous RNA (ceRNA), “sponging” miRNA off its cognate targets [26]. Subsequent studies have revealed that SNHG16 competitively binds with miR-4518 [33], miR-140-5p [34], miR-4500 [35] and miR-302a-3p [36] in various cancer types. Other confirmed mechanisms for lncRNAs in the cytoplasm involve post-transcriptional regulating mRNA stability or accessibility to the translational machinery [37], which is part of the interaction with RNA-binding proteins (RBPs) [38, 39]. For instance, lncRNA MEG3 induced Shp mRNA decay by interacting with PTBP1 to facilitate cholestatic liver injury [40]. Linc01093 triggered the mRNA decay of GLI1 through interaction with IGF2BP1 to suppress hepatocellular carcinoma (HCC progression [41].

In this study, we found that SNHG16 was mostly located in the cytoplasm in ESCC cell lines, which suggests its vital role in post-transcriptional regulation. We explored whether some RBPs interact with SNHG16 by RNA protein pull down and LC–MS analysis. According to the literature, EIF4A3 is one of the three core components of the exon junction complex (EJC), which causes mRNA decay and regulates protein expression at the translational and post-translational levels [42, 43]. We initially noticed that EIF4A3 was one of the RBPs interacting with SNHG16 and further confirmed the abundant binding relationship between them through RNA pull-down and RIP assays. Therefore, we hypothesized that SNHG16–EIF4A3 could regulate target mRNA stability. According to RNA-Seq, we found that RhoU was the common target of SNHG16 and EIF4A3. As expected, knocking down the expression of SNHG16 or EIF4A3 separately decreased the RhoU mRNA stability, and co-reduction of SNHG16 and EIF4A3 further decreased the half-life of RhoU. However, overexpression of SNHG16 failed to increase the mRNA stability of RhoU, which suggests that the mRNA stability regulation of SNHG16 was more dependent on EIF4A3.

Rho GTPases are class of small G proteins belonging to the Ras superfamily that regulate number of cell functions, including cell migration, cell proliferation, cell junction and cell polarity. The atypical Rho GTPase RhoU was originally isolated as a gene transcriptionally upregulated in wnt-1-transformed mouse mammary epithelial cells that shares distinct homology with Cdc42, as well as some biological functions [44]. For example, RhoU binds and activates p21-activated kinase (PAK1), induces filopodia and regulates cell tight junctions [45]. Impaired RhoU activity in fatty acid synthase-depleted cells leads to reduced adhesion turnover downstream of paxillin serine phosphorylation, which is rescued by the addition of exogenous palmitate [46]. Previous studies reported that knockout of RhoU led to increased peripheral adhesions and reduced paxillin S272 phosphorylation, which is required for adhesion disassembly [45]. Upregulated RhoU in prostate cancer correlated with disease progression, and silencing of RhoU was shown to reduce the migratory ability of MDA-MB-231 and PC3 breast cancer cells [45, 47]. In our study, we found that EIF4A3 could interact with RhoU, and the enrichment of RhoU in anti-EIF4A3 precipitates was strengthened by SNHG16 overexpression. In conclusion, we deemed that SNHG16 modulates RhoU expression by recruiting EIF4A3 to enhance the mRNA stability of RhoU.

To determine whether RhoU participates in the oncogenic role of SNHG16, we restrained the level of RhoU in SNHG16-overexpressing cells. Excitingly, we noticed that RhoU knockdown repressed SNHG16 carcinogenesis in ESCC cells. As we did not find an effective specific siRNA targeting RhoU, we were unable to perform in vivo experiments to confirm the SNHG16–RhoU axis. Despite this, we perceived the involvement of RhoU in SNHG16-induced ESCC cell proliferation and metastasis on the basis of in vitro studies.

## Conclusion

Our work demonstrates that upregulation of SNHG16 could promote ESCC growth and metastasis associated with tumour differentiation and T stage, which might be recognized as a potential therapeutic target for ESCC. Mechanistically, SNHG16 could interact with EIF4A3 and regulate RhoU mRNA stability. Targeting SNHG16–EIF4A3–RhoU signalling may provide new insights into ESCC treatment strategies. Our findings may provide new insight into how SNHG16 regulates mRNA stability and promote our comprehension of lncRNA regulatory characteristics in ESCC malignant development and progression.

## Supplementary Information

**Additional file 1: Figure S1. **EIF4A3 was upregulated in ESCC and promoted ESCC cell proliferation and migration. (A) Relative expression of EIF4A3 in human oesophageal cancer tissues (n = 162) compared with noncancerous tissues (n = 11) and a positive correlation with SNHG16 via the GEPIA database. (B) Western blot analysis of EIF4A3 after si-NC or si-EIF4A3 transfection in ESCC cells. Mock was the blank control group. GAPDH was used as an internal control. CCK-8 assays (C) and colony formation assays (D) were used to determine the proliferation ability of si-EIF4A3-transfected KYSE30 and KYSE410 cells. Transwell assays (E) and wound healing assays (F) were performed to investigate the migratory abilities of si-EIF4A3-transfected KYSE30 and KYSE410 cells. *P < 0.05, **P < 0.01, ***P < 0.001.

## Data Availability

Public data used in this work can be acquired from the GEPIA (GEPIA2, http://gepia2.cancer-pku.cn/#analysis), TCGA Research Network portal (https://portal.gdc.cancer.gov/) and Gene Expression Omnibus (GEO, https://www.ncbi.nlm.nih.gov/geo/). The raw data of our RNA-seq were available via the Gene Expression Omnibus (GEO) website (GSE189830). Other data used and analysed during this study are available from the corresponding author by reasonable request.

## Abbreviations

ESCC: Oesophageal squamous cell carcinoma
EC: Oesophageal cancer
LncRNA: Long noncoding RNA
ncRNAs: Noncoding RNAs
IEN: Intraepithelial neoplasia
RBP: RNA binding protein
ESD: Endoscopic submucosal dissection
ceRNA: Competing endogenous RNA
RIP: RNA immunoprecipitation
LC–MS: Liquid chromatography mass spectrometry
FISH: Fluorescence in situ hybridization
