# Supplementary material for: LncRNA SNHG16 promotes development of oesophageal squamous cell carcinoma by interacting with EIF4A3 and modulating RhoU mRNA stability
Source: Cell Mol Biol Lett. 2022 Oct 11;27:89. doi: 10.1186/s11658-022-00386-w (PMC9552503; doi:10.1186/s11658-022-00386-w)

Additional file 1 Table S1. Primer sequences

|  | Forward primer (5’-3’) | Reverse primer (5’-3’) |
| --- | --- | --- |
| sh-NC | TTCTCCGAACGTGTCACGT | |
| sh-SNHG16 #1 | GCTGCTAATTGTTCCTCCTCTAAA | |
| sh-SNHG16 #2 | GGCTTCATCTTTAAAGGAATG | |
| sh-SNHG16 #3 | GGAAGAGCCTAAGAGGAAACT | |
| si-NC | UUCUCCGAACGUGUCACGUTT | ACGUGACACGUUCGGAGAATT |
| si- EIF4A3 #1 | GCAAUCCAGCAACGAGCAATT | UUGCUCGUUGCUGGAUUGCTT |
| si- EIF4A3 #2 | CCAAUGUUGGCGAGGACAUTT | AUGUCCUCGCCAACAUUGGTT |
| si- EIF4A3 #3 | GCCGAUGAACGUUGCUGAUTT | AUCAGCAACGUUCAUCGGCTT |
| si- RHOU #1 | GACCUCUGGUUAAUUUAUATT | UAUAAAUUAACCAGAGGUCTT |
| si- RHOU #2 | CCUCUAAUCUGGAUGUUAATT | UUAACAUCCAGAUUAGAGGTT |
| si- RHOU #3 | GCUACACCAACACAGACAUTT | AUGUCUGUGUUGGUGUAGCTT |
| NELL2 | GCATTACCGCTCAGGCAGTCAC | AGAGCTTGTGCCACTTGTCATCAG |
| SNHG16 | TGTTTCGTTTCTGGTGACTGA | TTGATGACTACACGGCTTTGC |
| GAPDH | CGGAGTCAACGGATTTGGTCGTAT | AGCCTTCTCCATGGTGGTGAAGAC |
| STAGALNAC1 | CAGGACACAAAGACGACCCAAGG | CTTTGCCCTGGTGCTTCTCTGAC |
| FOXO6 | AGGATAAAGGCGACAGCAACAGC | TTCAGCATCCACCACGAACTCTTG |
| EIF4A3 | GCTGCTTGCTCTCGGTGACTAC | GCTTCCTGATGTCCTCGCCAAC |
| P4HTM | TATTTGAACAACGTCACTGGTG | CGTCATCCTGAATCAGACTCAT |
| AGR2 | AGAGATACCACAGTCAAACCTG | CTTTAAAGCTTGACTGTGTGGG |
| WNT4 | GAACCTGGAAGTCATGGACTC | GAAGAGATGGCGTACACGAAG |
| RHOU | GGATGAATTTGACAAGCTGAGG | TGCATTTGTCCAACTCAATGAG |

Table S2. Clinical specimen information

| Sample | Gender | Age (year) | Tumor size (cm) | Tumor differentiation | Clinical stage |
| --- | --- | --- | --- | --- | --- |
| 1 | Male | 61 | 6*3*1.2 | High | II |
| 2 | Female | 53 | 4.5*3.5*1.5 | High | III |
| 3 | Male | 68 | 6*5*0.5 | Moderate | III |
| 4 | Male | 75 | 5.5*3.5*0.1 | Moderate | I |


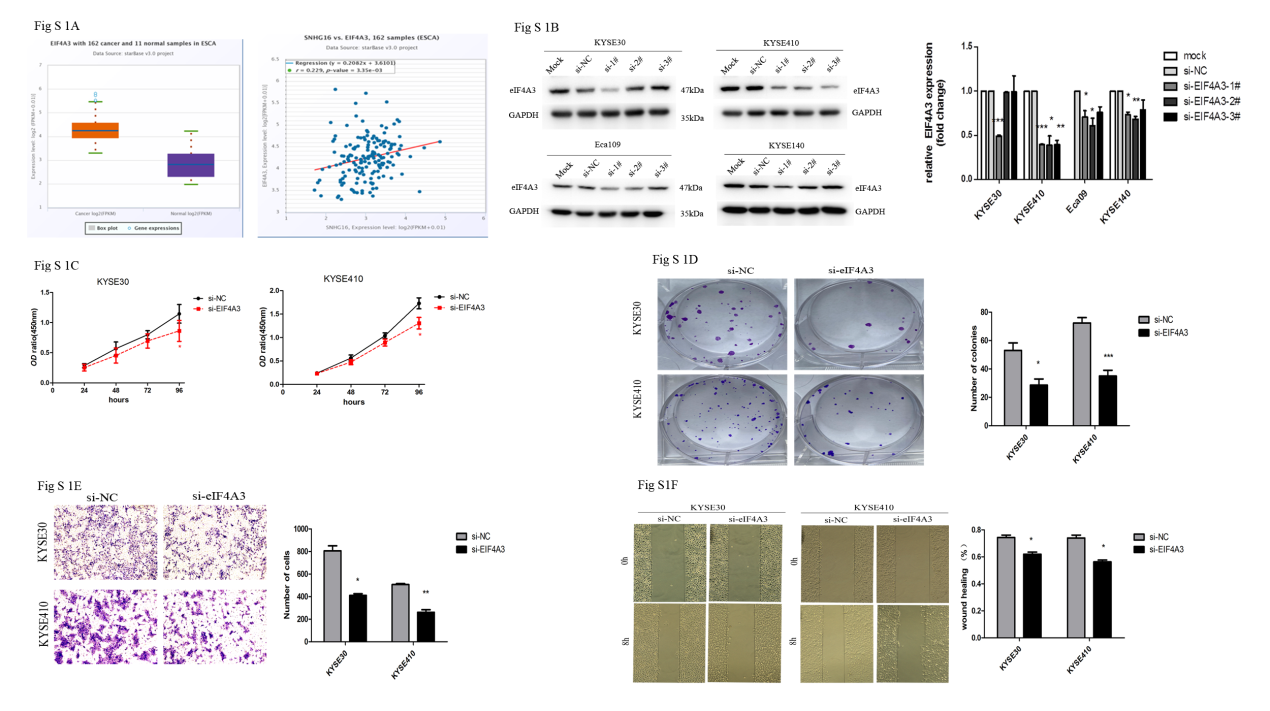


Supplemental material wb-gels


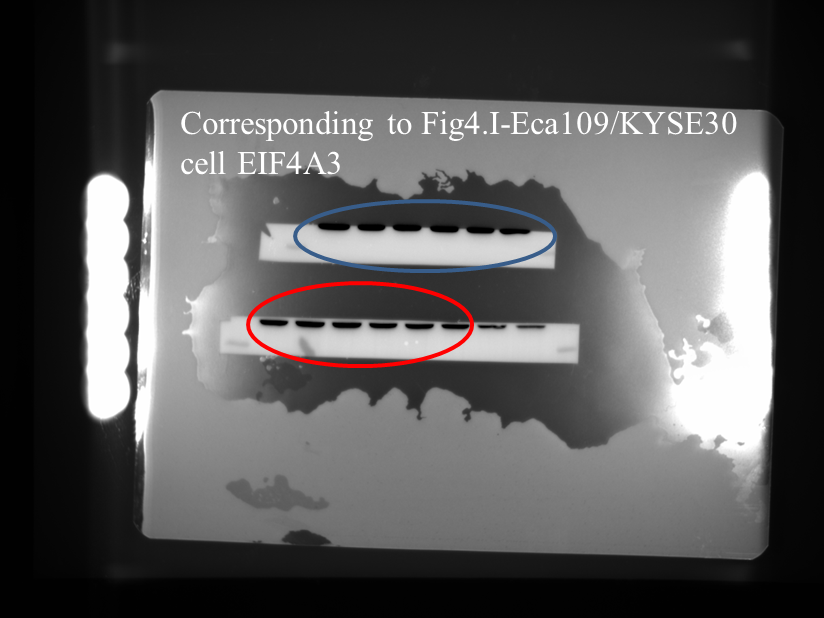


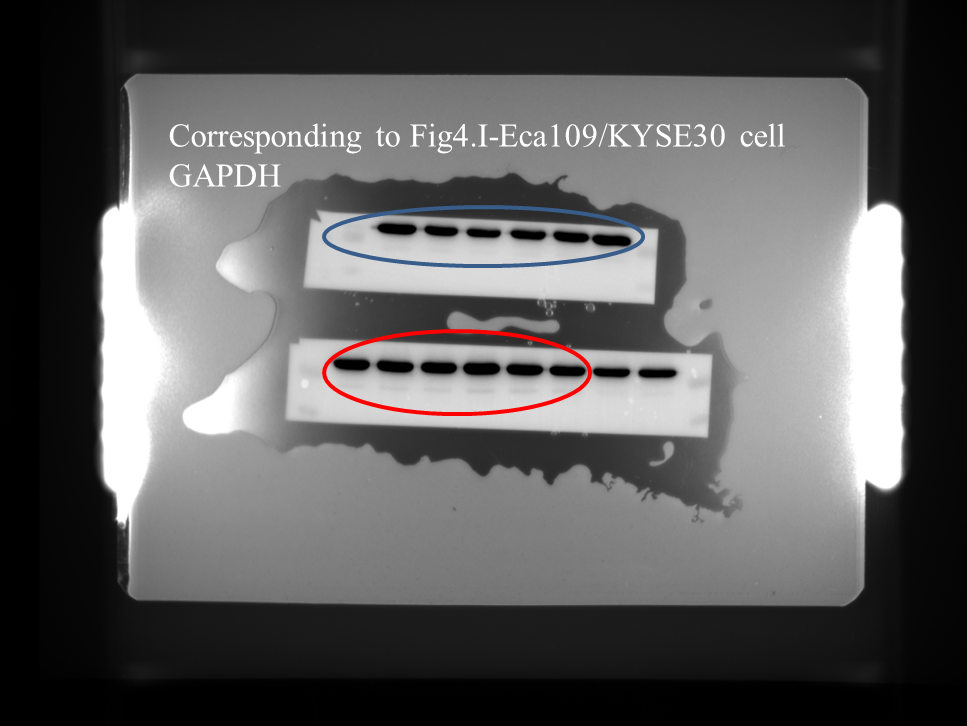


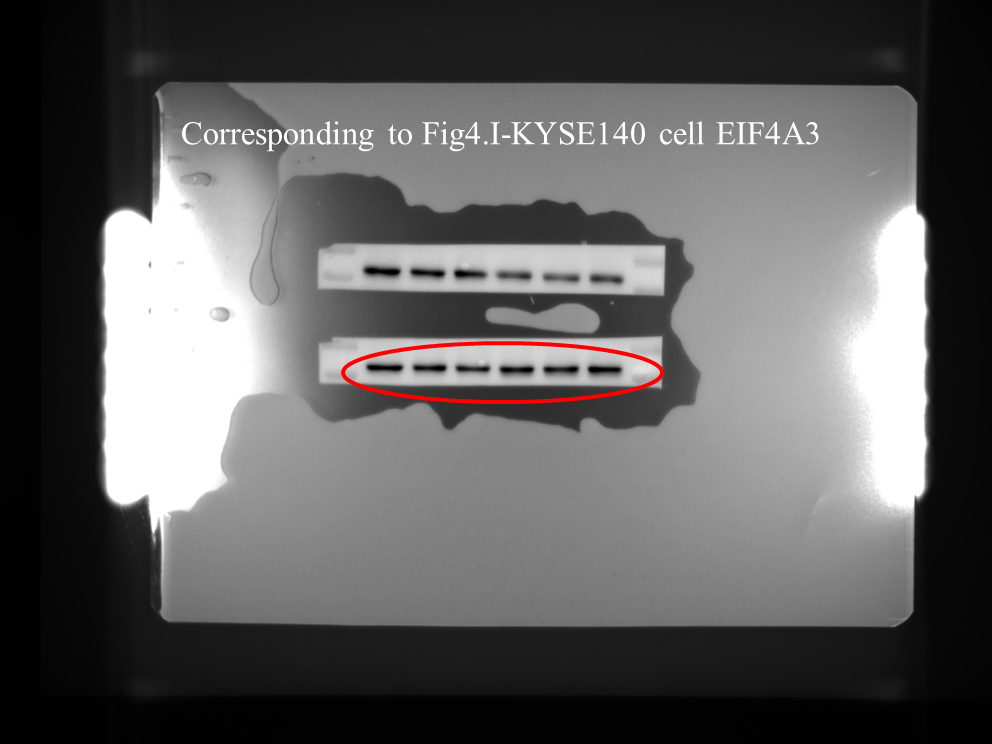


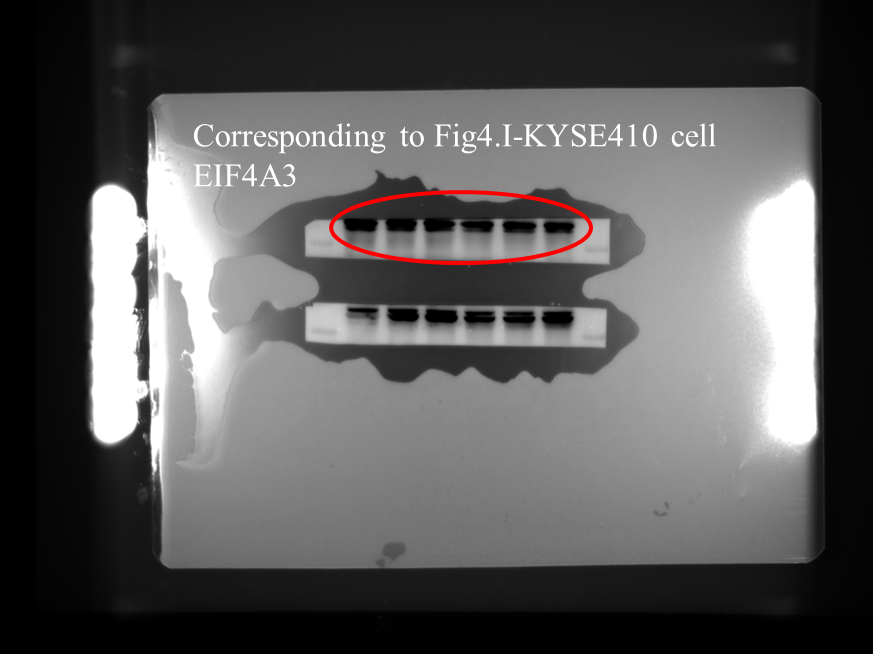


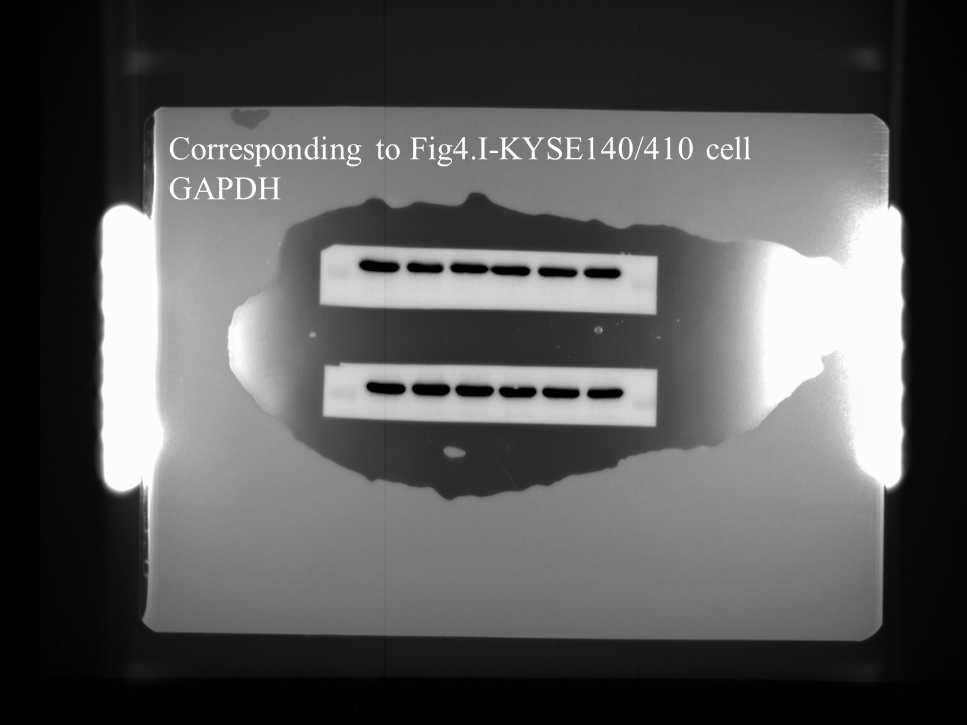


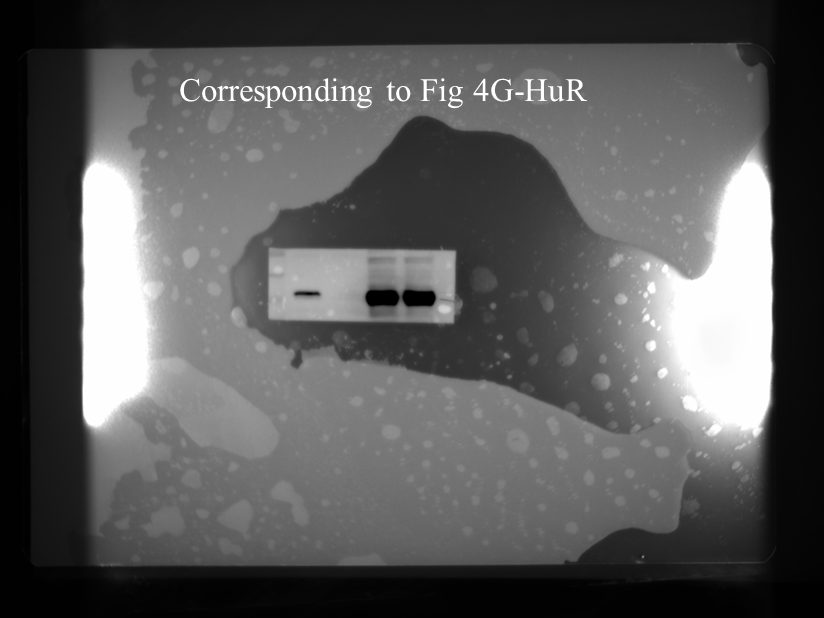


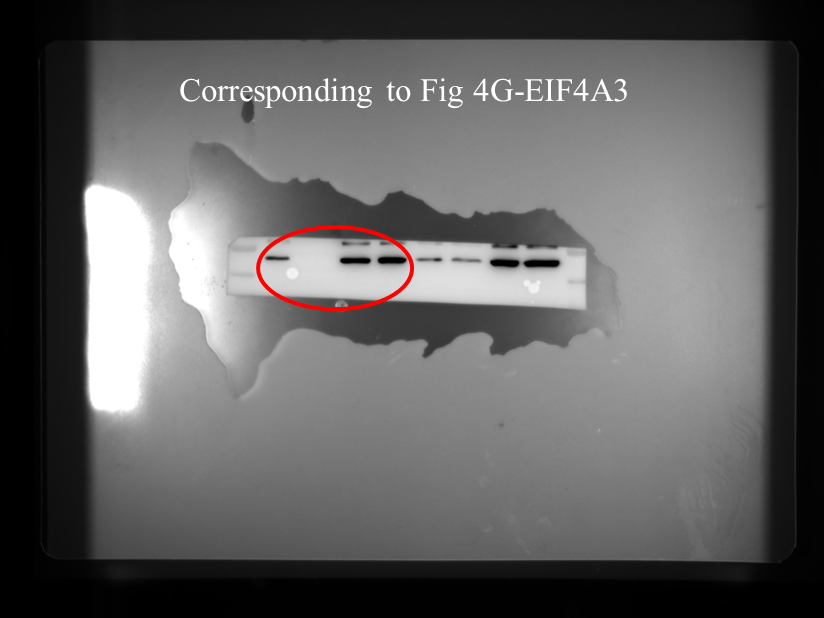


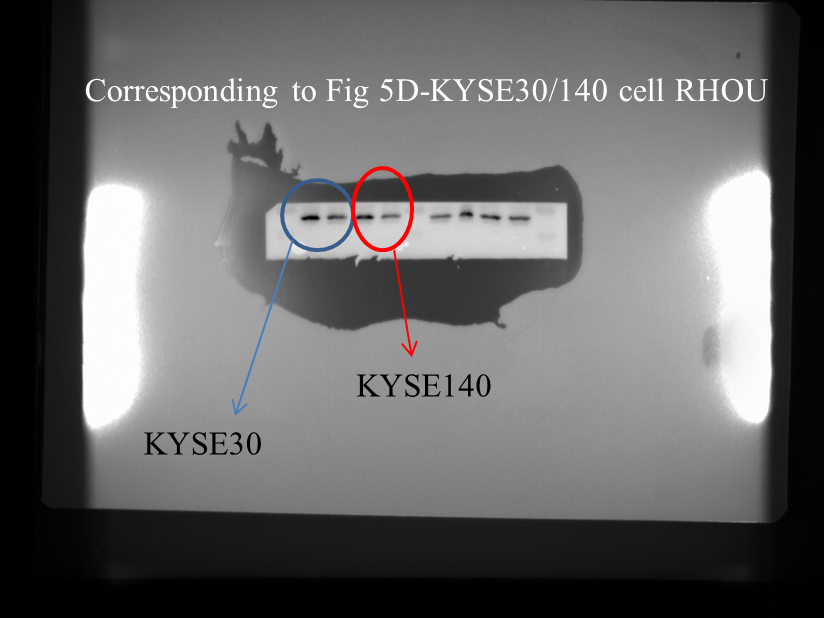


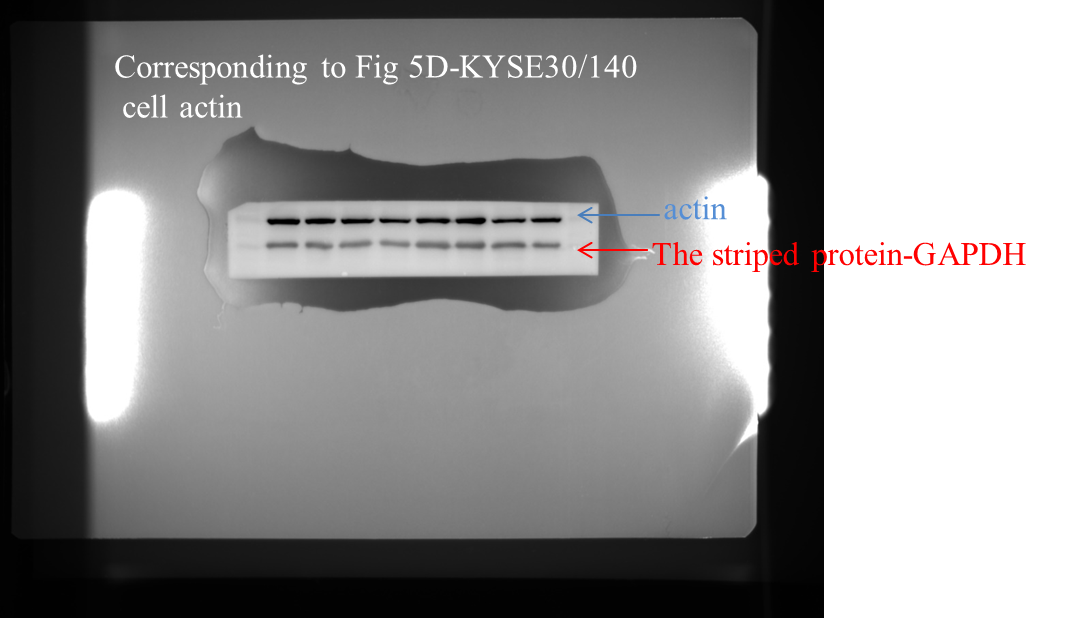


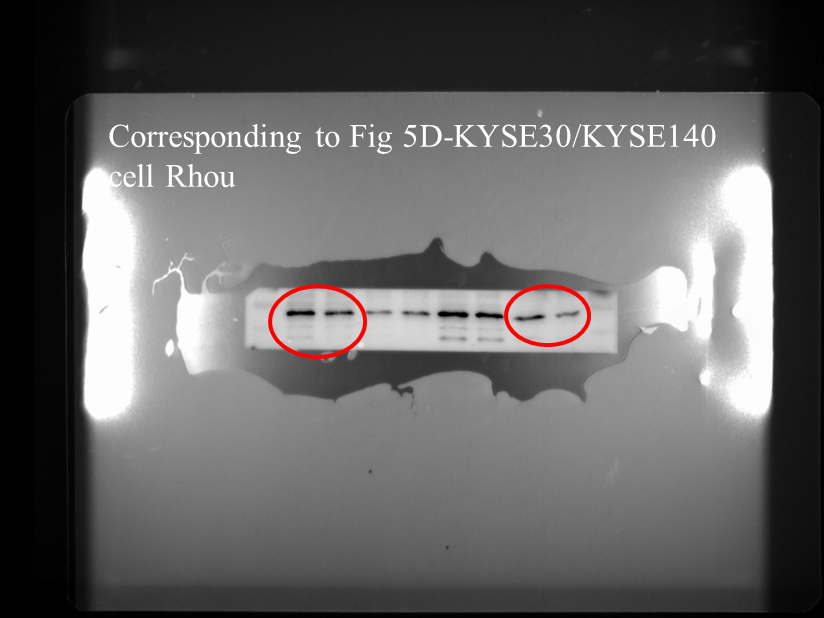


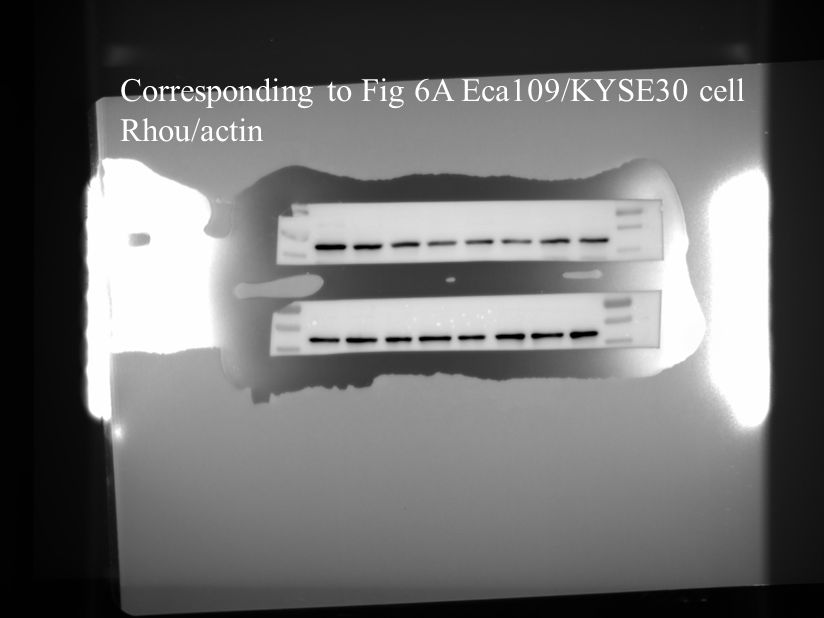


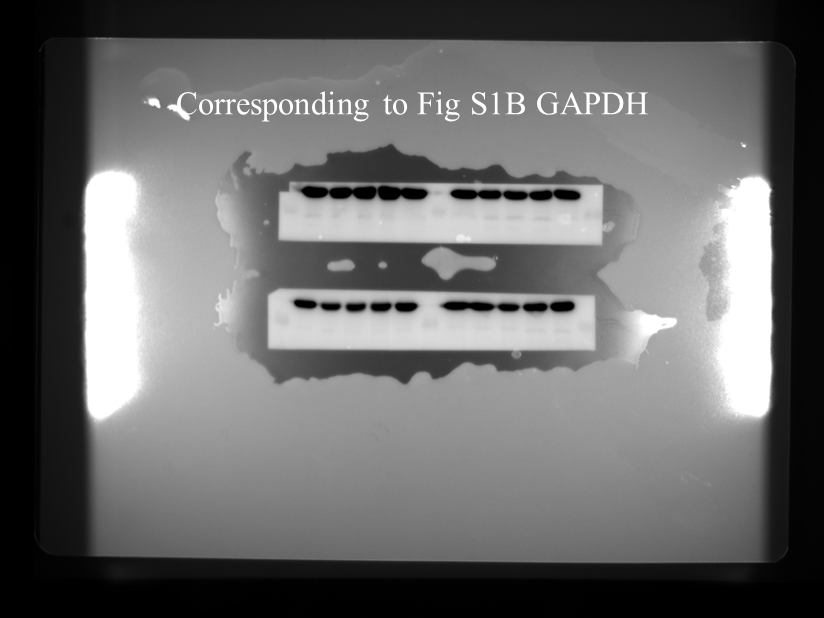


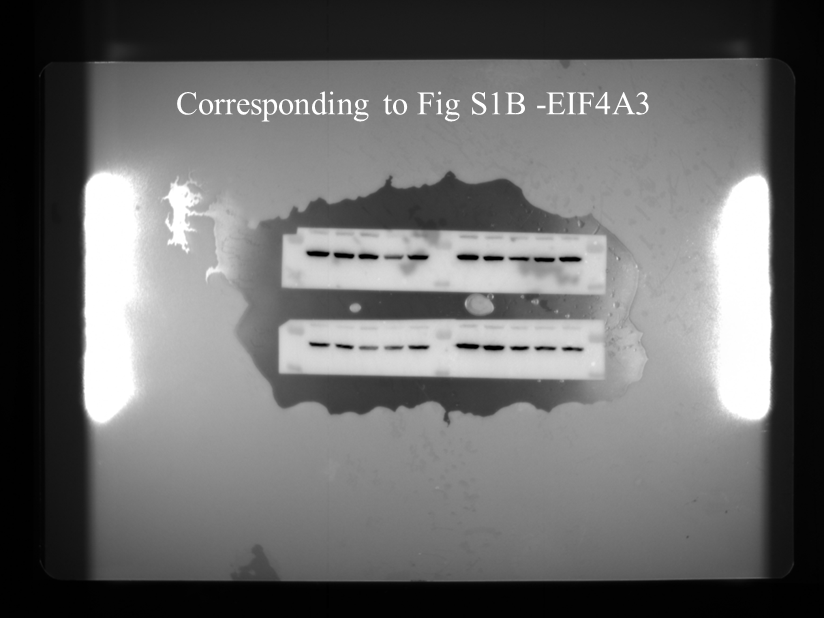

Supplement: Supplementary file 1 — Additional file 1: Figure S1. EIF4A3 was upregulated in ESCC and promoted ESCC cell proliferation and migration. (A) Relative expression of EIF4A3 in human oesophageal cancer tissues (n = 162) compared with noncancerous tissues (n = 11) and a positive correlation with SNHG16 via the GEPIA database. (B) Western blot analysis of EIF4A3 after si-NC or si-EIF4A3 transfection in ESCC cells. Mock was the blank control group. GAPDH was used as an internal control. CCK-8 assays (C) and colony formation assays (D) were used to determine the proliferation ability of si-EIF4A3-transfected KYSE30 and KYSE410 cells. Transwell assays (E) and wound healing assays (F) were performed to investigate the migratory abilities of si-EIF4A3-transfected KYSE30 and KYSE410 cells. *P < 0.05, **P < 0.01, ***P < 0.001. [file 11658_2022_386_MOESM1_ESM.docx]
